# The opposing roles of lethal and nonlethal effects of parasites on host resource consumption

**DOI:** 10.1002/ece3.9973

**Published:** 2023-04-13

**Authors:** Emlyn J. Resetarits, William T. Ellis, James E. Byers

**Affiliations:** ^1^ Odum School of Ecology University of Georgia Athens Georgia 30602 USA; ^2^ Center for the Ecology of Infectious Diseases University of Georgia Athens Georgia 30602 USA; ^3^ Marine Institute University of Georgia Darien Georgia 31305 USA

**Keywords:** *Helisoma trivolvis*, mortality, parasite‐mediated effects, temperature, trematoda, trophic cascade

## Abstract

Although parasites can kill their hosts, they also commonly cause nonlethal effects on their hosts, such as altered behaviors or feeding rates. Both the lethal and nonlethal effects of parasites can influence host resource consumption. However, few studies have explicitly examined the joint lethal and nonlethal effects of parasites to understand the net impacts of parasitism on host resource consumption. To do this, we adapted equations used in the indirect effects literature to quantify how parasites jointly influence basal resource consumption through nonlethal effects (altered host feeding rate) and lethal effects (increased host mortality). To parametrize these equations and to examine the potential temperature sensitivity of parasite influences, we conducted a fully factorial lab experiment (crossing trematode infection status and a range of temperatures) to quantify feeding rates and survivorship curves of snail hosts. We found that infected snails had significantly higher mortality and ate nearly twice as much as uninfected snails and had significantly higher mortality, resulting in negative lethal effects and positive nonlethal effects of trematodes on host resource consumption. The net effects of parasites on resource consumption were overall positive in this system, but did vary with temperature and experimental duration, highlighting the context dependency of outcomes for the host and ecosystem. Our work demonstrates the importance of jointly investigating lethal and nonlethal effects of parasites and provides a novel framework for doing so.

## Graphical abstract

In this article, we jointly investigate the lethal and non‐lethal effects of trematode parasites on host resource consumption across three temperatures. To do this we experimentally investigated the impacts of parasites on the survivorship and feeding rates of naturally infected, lab‐reared snails. We then modified indirect effects equations and parameterized them with experimental data to calculate the lethal and nonlethal effects. We found that non‐lethal effects (green) increased resource consumption through increased host feeding rate, while lethal effects (blue) decreased consumption through increased host mortality. These effects were partially offsetting and resulted in a small net increase on host resource consumption caused by parasites, but only at certain temperatures.

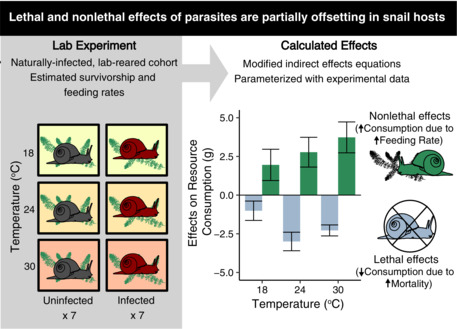

## 1 INTRODUCTION

Over the past 10 years, there has been great effort to place parasites into food webs (Byers, 2009; Hatcher et al., 2012; Lafferty et al., 2008) and broader ecological theory (Buck & Ripple, 2017; Daversa et al., 2021; Dunn et al., 2012; Raffel et al., 2008; Thomas et al., 2005). Specifically, recent work has drawn parallels between parasite–host and predator–prey literature such as trophic cascades, indirect effects, and nonlethal effects (Anaya‐Rojas et al., 2019; Buck, 2019; Buck & Ripple, 2017; Daversa et al., 2021; Dunn et al., 2012; Raffel et al., 2008). Parasites, like predators, can have lethal effects on their hosts, as well as cause trait responses in their hosts that result in nonlethal effects. These host trait responses can occur to avoid contact with parasites (i.e., avoidance behaviors, risk‐induced trait responses, nonconsumptive effects; e.g., Koprivnikar et al., 2021; Weinstein et al., 2018), to resist active infection, or as a response to being infected by the parasite (Buck, 2019; Daversa et al., 2021). Such trait responses caused by infection include changes in host physiology (Bernot et al., 2008), behavior (Mouritsen & Poulin, 2005; Sato et al., 2012), and feeding rates (Bernot et al., 2008; Hernandez & Sukhdeo, 2008; Morton & Silliman, 2020; Sánchez et al., 2016; Wood et al., 2007). For example, the common periwinkle, *Littorina littorea*, decreases macroalgal consumption when infected with trematode parasites, resulting in a positive indirect effect of the parasite on the macroalgae (Wood et al., 2007).

Lethal and nonlethal effects of parasites do not work in isolation, but rather, can act jointly on hosts and indirectly impact other species in the community such as primary producers and can alter basal resources. Despite this importance, little work has investigated the relative roles of lethal and nonlethal effects of parasites on resource consumption (Walsman et al., 2022). Lethal effects should always reduce host density in the short term and thus decrease consumption on basal resources. Therefore, the direction and magnitude of nonlethal effects will determine whether net effects of a parasite are positive or negative. This is important because effects could compound if parasites increase host mortality and decrease host feeding rate or could balance each other out if parasites increase both host mortality and host feeding rate.

We used a guild of larval trematode parasitic castrators and their first intermediate snail hosts, *Helisoma trivolvis*, to investigate the relative importance of lethal and nonlethal effects of parasites on consumption of resources by hosts. Parasitic castrators are hypothesized to have some of the strongest nonlethal effects of all predator and parasite groups due to their strong fitness consequences and significant host longevity post‐infection (Daversa et al., 2021). *H. trivolvis* are freshwater pulmonate snails typically found on emergent vegetation in lakes and ponds, feeding on plant tissue, periphyton, and decaying matter. Trematodes increase host mortality in some snail taxa, which should result in reduced consumption of basal resources (e.g., Sorensen & Minchella, 1998). Trematodes also cause increased feeding rates in some snail species (Bernot et al., 2008; Muñoz et al., 2018), but decreased feeding rates in others (Morton & Silliman, 2020; Muñoz et al., 2018; Wood et al., 2007).

The direct effects of parasites on hosts also vary with temperature (e.g., Byers, 2020, 2021; Lafferty, 2009). Temperature is known to have strong effects on feeding rates (Kratina et al., 2012; O'Connor et al., 2009; Shurin et al., 2012) and metabolism (e.g., Brown et al., 2004). Previous work has found that temperature and infection status jointly influence nonlethal (Larsen & Mouritsen, 2009) and lethal effects (Fredensborg et al., 2005; Gehman et al., 2018) on infected aquatic ectotherm hosts. For example, Larsen and Mouritsen (2009) found that at higher temperatures associated with predicted warming scenarios, the reduced feeding rate of infected periwinkle snails and associated positive indirect effects on macroalgae seen by Wood et al. (2007, described above) disappeared entirely. While it is well understood that temperature should influence both mortality and feeding rate, it remains unclear how temperature‐dependent changes in these rates might compare, and what that means for total consumption by hosts across a range of temperatures.

Overall, we quantify and parse the lethal and nonlethal effect of parasites on host consumption by modifying equations used to examine density and trait‐mediated indirect effects of predators and applying these equations to experimental data on the impacts of parasites on host survival and feeding rate. To accomplish this, we first quantify mortality and feeding rate of infected and uninfected hosts from the same cohort (allowing us to control for age and environment) across three temperatures through a lab experiment. We then extract estimates of survival duration (from the mortality data) and feeding rate to calculate the lethal and nonlethal impacts of parasites on host consumption across the duration of our experiment (65 days). For these calculations, we modify equations developed to examine density and trait‐mediated indirect effects of predators (Okuyama & Bolker, 2007), which are analogous to lethal and nonlethal effects, respectively. We then combine the lethal and nonlethal effects to estimate net host consumption (during our 65‐day experiment) for infected and uninfected hosts to understand the cumulative impacts parasites have. Using this approach we address two questions: (1) What is the relative importance of lethal and nonlethal effects of parasites on basal resource consumption? (2) How do lethal, nonlethal, and their net effects on resource consumption change with temperature?

## 2 METHODS

### 2.1 Estimating feeding rates and survivorship

To maximize the chance that differences in feeding rate and mortality were due to parasitism and not an underlying genetic difference that increased infection likelihood, we used lab‐reared snails. Four to six adult snails were collected from each of three ponds at the Huie Constructed Wetlands in Clayton County, GA (33°29′N, 84°18′W). In the lab, snails from each pond were allowed to lay eggs for three days (November 2, 2019 to November 5, 2019). Eggs were reared in the lab under constant conditions. Once greater than six mm long, lab‐reared snails were individually marked, placed in modified plastic minnow trap enclosures (15 snails per trap), and set out across nine ponds (all within 1.3 km radius) at the Huie Wetlands for three months (April 27, 2020 to July 22, 2020) to ensure some snails became infected. Snails were brought back to the lab and individually housed in 160 mL plastic cups with freshwater media and agar food cubes under fluorescent lights for nine weeks. Water changes were done weekly, and snails were checked twice a week for cercariae (free‐swimming parasite stage) or egg masses. The fluorescent lights created a bright, warm environment that prompted trematodes to release cercariae from infected snails (a process called shedding). Snails that shed cercariae were marked as infected. Snails with mature trematode infections are castrated and cannot produce eggs. Those that did not shed but did lay eggs were marked as uninfected, since ramshorn snails are hermaphroditic and can store sperm for over 16 weeks, allowing them to produce eggs in isolation (Norton & Newman, 2016). Snails that neither shed nor produced eggs were not included in the experiment because their infection status could not be definitively verified nondestructively. Once infected, snails typically cannot clear the infection, and therefore there should be no resistant snail class. Infected snails displayed three morphotypes of cercariae: a magnacauda type (*n* = 2), putative strigea (*n* = 14) type, and one unidentified (*n* = 3, Table S2). All three morphotypes were used for analysis to estimate the cumulative effects of this trematode guild. We also performed identical analyses on the dominant cercarial morphotype (putative strigea), to investigate species‐specific effects on the snail host (see Supplementals).

Infected and uninfected *H. trivolvis* were paired based on shell length (mean size = 20.27 ± 0.17 mm, mean difference = 0.614 mm) to ensure sizes were well distributed between treatments. Each pair (*n* = 21) of *H. trivolvis* was then randomly assigned to one of three temperature treatments (18, 24, and 30°C) that were within the temperature range they experienced during the summer at the Huie Constructed wetlands, with 24°C being the mean temperature (unpublished). For each temperature, seven infected and seven uninfected *H. trivolvis* were housed individually in 300 mL, 56.6 mm diameter, Freund 5023B06‐B spice jars filled with media. Media was composed of tap water, with BetaSafe to eliminate chlorine and a calcium supplement (Seachem Laboratories Inc, Equilibrium™) to promote shell growth. Jars were kept in water baths and temperatures were controlled using an Inkbird Digital Outlet Heat Temperature Controller throughout the experiment (65 days). Two infected snails died before the experiment started (*n* = 6 at 18°C and 30°C) and were not included in analyses.

Because sample sizes were relatively low (6 or 7 individuals per infection status per temperature treatment), we boosted replication of feeding rate trials by using a repeated measures design. Specifically, we conducted three shorter term trials within the 65‐day mortality experiment. All *H. trivolvis* were starved for two days prior to feeding trials. At the start of each trial they were each given an agar food cube. Agar food cubes are composed of a homogenized liquid of 4 g organic Nutrena Naturewise® Meatbird 22% protein crumble (a.k.a. chicken feed) and 2 g Omega One Fishwater Flakes dissolved and strained in 150 mL media and mixed with 1.5 g agarose to gelatinize the mixture. This mixture was poured across three petri dishes and allowed to cool before being cut into cubes and weighed. These cubes are suitable for an omnivorous diet, can be easily standardized and quantified, and do not degrade in water (loss over 5 days = 0.02 ± 0.004 g). This allowed us to accurately quantify the effect of infection status on snail feeding rates.

Food cube masses were measured and recorded prior to trials (weight = 0.617 ± 0.009 g). Each temperature treatment received two control jars containing only food and media to account for changes in mass over the trial period. Feeding trials ran for five days. Food cubes were removed from each jar, placed on a paper towel to absorb excess water, and measured for wet mass again. After each trial, dead snails were removed and excluded from that and subsequent trials. We used the same *H. trivolvis* in three consecutive trials (September 24, 2020, October 1, 2020, October 8, 2020) giving us repeated measures of consumption for each snail. We recorded dates of mortality of all snails throughout the entire duration of the experiment. The experiment was ended on November 27, 2020.

### 2.2 Statistical methods

Food cube decomposition for each temperature and trial combination was determined by subtracting post‐experiment mass from pre‐experiment mass for each of two controls and averaging them. Daily feeding rates for each snail were determined by subtracting the post‐experiment food cube mass from the pre‐experiment mass minus the associated intrinsic decomposition and dividing this total by five days. Nine snails had consumption values that were less than 0 (min = −0.027 g) likely due to small measurement errors, and these values were bounded to 0 to make biological sense. Using R, we used a linear mixed effects model to investigate feeding rate by *H. trivolvis* as a function of infection status, temperature, the interaction between temperature and infection status, initial snail size, and experimental trial (1–3) and a random effect of snail ID, since this was a repeated‐measures design (Bates et al., 2015; R Core Team, 2020).

To investigate survivorship in *H. trivolvis*, we used the R packages “survival” and “survminer” to fit a Cox proportional hazards regression model including infection status and temperature treatments (Kassambara et al., 2020; Therneau, 2020). Because there was no variation in mortality for uninfected snails, we coded one live snail as dead (at day 56) from each temperature in order to provide a nonzero variability that could allow statistical calculations. We did not include an interaction term because no uninfected snails died. This coding of one data point was done only for statistical purposes and was not used for visual presentation of the data or for estimating survivorship duration (see below). To examine how robust our results were to trematode identity, we also ran these analyses using only the most common morphotype (putative strigea) (see Supplement for details).

### 2.3 Calculating lethal and nonlethal effects across temperatures

To determine how changes in mortality and feeding rate due to parasitism jointly influence the snail's total resource consumption and how these processes vary with temperature, we modified equations used to estimate trait‐mediated (TMIE) and density‐mediated indirect effects (DMIE) in predator–prey systems (Peacor & Werner, 2001; see Equation 2 from Okuyama & Bolker, 2007). Because parasites (as defined by Lafferty & Kuris, 2002) attack only one host during a given life stage, whereas predators generally attack multiple prey, we estimated effects of parasites at the individual‐host level rather than the population level, typically used for predators. This means that our lethal effects on consumption (analogous to DMIE) are based on the number of days an individual survives, rather than the number of individuals that survive an experiment of a given duration.

To estimate nonlethal (Equation 2 below) and lethal (Equation 3 below) effects of trematode infection on basal resource consumption, we used our empirical data of survival duration (days) and feeding rate (g/day; Table S1). We used the emmeans package in R to calculate estimated marginal means and standard error for average per‐capita feeding rate (g/day) for infected $\left(\bar{F\_I_{t}}\right)$ and uninfected snails $\left(\bar{F\_U_{t}}\right)$ for each temperature, t. We also calculated mean and standard error for survival duration for infected $\left(\bar{S\_I_{t}}\right)$ and uninfected snails $\left(\bar{S\_U_{t}}\right)$ at temperature t (Table S1; Figure S2c). By multiplying per‐capita feeding rate (g/day) by survival duration (days), we could estimate total resource consumption (g) at temperature t over the duration of the experiment (d = 65 days) for infected ($\bar{S\_I_{t}}\cdot \bar{F\_I_{t}}$) and uninfected ($\bar{S\_U_{t}}\cdot \bar{F\_U_{t}}$) snails. Our models assume that the feeding rate was constant over time, which is supported by our feeding trials (Figure S1), and no exhaustion of resources by snails.

Lethal (LE) plus nonlethal (NLE) effects should explain the difference between total resource consumption for infected and uninfected snails:

(1) $$\bar{S\_I_{t}}\cdot \bar{F\_I_{t}}=\bar{S\_U_{t}}\cdot \bar{F\_U_{t}}+\left(\mathrm{LE}+\mathrm{NLE}\right)$$

where $\bar{S\_I_{t}}$ and $\bar{S\_U_{t}}$ are the average number of days infected and uninfected snails survived at temperature *t*, and $\bar{F\_I_{t}}$ and $\bar{F\_U_{t}}$ are the average per‐capita feeding rate of infected and uninfected snails at temperature *t*. We can estimate nonlethal effects (NLE) of parasites on feeding rate as the survival duration of uninfected snails multiplied by the difference between infected and uninfected host feeding rates:

(2) $$\mathrm{NLE}=\bar{S\_U_{t}}\left(\bar{F\_I_{t}}-\bar{F\_U_{t}}\right)$$

We use survival duration of uninfected snails $\left(\bar{S\_U_{t}}\right)$ to calculate NLE to isolate the effect of parasites on feeding rate from the effect on survival duration. If $\bar{F\_I_{t}}>\bar{F\_U_{t}}$, parasites increase total resource consumption by the host. By plugging in Equation (2) into Equation (1) (see supplementals for derivation), we see that lethal effects are equal to the feeding rate of infected snails multiplied by the difference in survival duration between infected and uninfected snails:

(3) $$\mathrm{LE}=\bar{S\_I_{t}}\cdot \bar{F\_I_{t}}-\bar{S\_U_{t}}\cdot \bar{F\_I_{t}}=\bar{F\_I_{t}}\left(\bar{S\_I_{t}}-\bar{S\_U_{t}}\right)$$

Here, our calculations of lethal effects (LE) estimate the difference between survival duration of infected and uninfected hosts when including the effect of infection on feeding rate; calculating LE and NLE in this way avoids double counting any parasite effects of mortality or consumption (see Supplement for additional details).

We calculated NLE and LE separately for each of our three temperatures (Equations 2 and 3). We then estimated the net effects of parasitism on resource consumption $(\bar{S\_I_{t}}\cdot \bar{F\_I_{t}}$) – $\left(\bar{S\_U_{t}}\cdot \bar{F\_U_{t}}\right)$ at each temperature by summing LE and NLE:

(4) NetConsumption Effects=LE+NLE

Finally, we calculated total resource consumption during the course of the experiment (65 days) for infected $\left(\bar{S\_I_{t}}\cdot \bar{F\_I_{t}}\right)$ and uninfected ($\bar{S\_U_{t}}\cdot \bar{F\_U_{t}}$) snails. Error was propagated using standard procedures and included to quantify uncertainty but was not used to estimate significance.

### 2.4 Investigating importance of experimental duration

Experimental duration likely influences the results of such analyses and experiments, since the difference in mortality between infected and uninfected snails increases over time. To demonstrate the importance of experimental duration on our results at each temperature, we estimated total resource consumption of infected $\left(\bar{S\_I_{t}}\cdot \bar{F\_I_{t}}\right)$ and uninfected ($\bar{S\_U_{t}}\cdot \bar{F\_U_{t}}$) snails by calculating $\bar{S\_I_{t}}$ and $\bar{S\_U_{t}}$ up to day 10 using the empirical data. We continued recalculating $\bar{S\_I_{t}}$ and $\bar{S\_U_{t}}$ [and subsequently, $\left(\bar{S\_I_{t}}\cdot \bar{F\_I_{t}}\right)$ and ($\bar{S\_U_{t}}\cdot \bar{F\_U_{t}}$)] at increasing 10‐day increments up to 100 days, as well as for the duration of our actual experiment (65 days). Predicting out past our actual experimental duration (65 days to 100 days), we assumed no additional mortality, since all infected snails had died by 65 days and no uninfected snails had yet died.

## 3 RESULTS

### 3.1 Estimating feeding rates and survivorship

**FIGURE 1:**
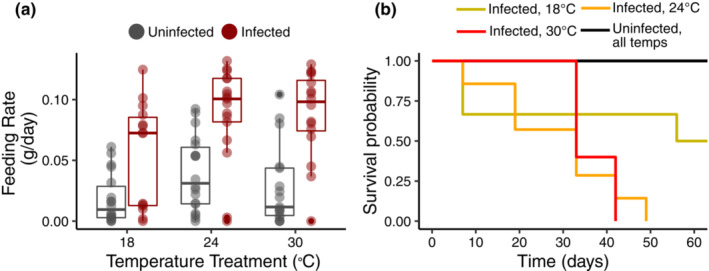
(a) Infection status and temperature significantly impact feeding rates. Infected snails (dark red) eat significantly more than uninfected snails (gray). Estimated marginal means and standard error were used to calculate feeding rates for uninfected and infected hosts at each temperature (*F*_*U*_*t*_ and *F*_*I*_*t*_) for calculating lethal and nonlethal effects. (b) Infection status and temperature also significantly affect survivorship. Survivorship is significantly lower for infected snails than uninfected snails. There was no mortality for uninfected snails at all temperatures (black line). Yellow = infected, 18°C; orange = infected, 24°C; red = infected, 30°C. Mean and standard error for survivorship duration at each temperature for infected and uninfected snails (*S*_*U*_*t*_ and *S*_*I*_*t*_) were used for calculating lethal and nonlethal effects.

Both temperature (𝜒^2^ = 7.141, df = 2, *p* = .028) and infection status (𝜒^2^ = 24.635, df = 1, *p*‐value < .0001) significantly affected snail feeding rate. On average, infected snails ate over twice as much as uninfected snails (0.073 ± 0.006 g/day vs 0.029 ± 0.006 g/day; Figure 1a), and feeding rate increased with temperature. There was no interaction between temperature treatment and infection status (𝜒^2^ = 1.577, df = 2, *p* = .454), nor an effect of experimental trial (𝜒^2^ = 2.144, df = 1, *p* = .143). Consumption decreased with snail size (𝜒^2^ = 4.919, df = 1, *p* = .027; Figure S2) irrespective of infection status.

Both temperature (z = 1.977, df = 2, *p* = .048) and trematode infection status (z = 4.021, df = 1, *p*‐value < .0001) significantly affected snail survivorship (Figure 1b). All infected snails at 24°C and 30°C died before the end of the experiment (65 days), and only half of the infected snails survived at 18°C. No uninfected snails died during the experiment. Feeding rate and survivorship results were qualitatively similar when focusing on only the most common trematode morphotype (putative strigea cercariae), except that temperature was not a significant factor for snail mortality, likely due to uneven and insufficiently low sample sizes for some of the temperatures when other morphotypes were removed (see supplementals for details).

### 3.2 Calculating lethal and nonlethal effects across temperatures

**FIGURE 2:**
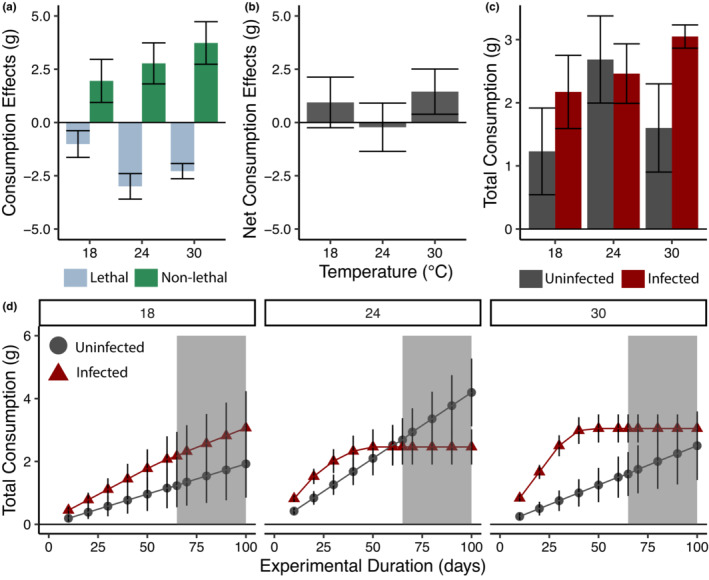
How parasites impact basal resource consumption. (a) Estimated lethal (LE) and nonlethal (NLE) effects of parasites on host resource consumption at each temperature treatment. Lethal effects decreased consumption (blue), while nonlethal effects (feeding rate) increased resource consumption (green). (b) Net consumption effects (NCE). Recall that NCE = LE + NLE (Equation 4). At 18°C and 30°C, parasites overall had a net positive impact on resource consumption, where at 24°C parasites have minimal effect on basal resources due to lethal and nonlethal effects being equal and in opposing directions. (c) Total basal resource consumption over the course of the experiment (65 days) for infected (dark red) and uninfected (gray) snails. (d) The effect of experimental duration (days) on cumulative consumption of basal resources by infected (red triangles) and uninfected (gray circles) snails. The gray area represents extrapolations beyond our experimental duration.

The relative roles of lethal and nonlethal effects varied across temperatures (Figure 2a). At 18°C, the magnitude of nonlethal effects was slightly greater than lethal effects, resulting in positive net effects on resource consumption (Figure 2b). At 24°C, increased mortality of infected snails resulted in nonlethal effects and lethal effects balancing each other out over the course of the experiment. At 30°C, despite high mortality of infected snails, nonlethal effects were greater than lethal effects resulting again in positive net effects on resource consumption. Over the course of the experiment, we estimated that an infected snail consumed 80% more basal resources than an uninfected snail at 18°C, 8% less at 24°C, and 90% more at 30°C (Figure 2c).

### 3.3 Investigating importance of experimental duration

We found that the net effects of parasites on resource consumption were sensitive to experimental duration, resulting in strong differences in total resource consumption between infected and uninfected snails only at certain experimental durations (Figure 2d). At 18°C, infected snails cumulatively consumed more than uninfected snails for all durations. At 24°C, infected snails cumulatively consumed more for short experimental durations. As duration extended, the increased feeding rate by infected snails became offset by increased mortality, such that at 65 days we found no difference in cumulative consumption. Projecting out past our data, our results suggest that total consumption may be greater for uninfected snails at longer experimental durations. At our highest temperature, 30°C, despite high mortality, infected snails cumulatively consumed more than uninfected snails for most experimental durations, with the maximum difference occurring around 40 days.

## 4 DISCUSSION

Here we demonstrate a method to estimate lethal and nonlethal effects of a guild of trematode parasites on host resource consumption. Using our experimental data as a test case, we found that lethal effects of the parasite resulted in decreased resource consumption and nonlethal effects (altered feeding rate) resulted in increased resource consumption. Lethal and nonlethal effects of parasites on host resource consumption in this system were opposing and partially offsetting. Importantly, our results demonstrate that temperature and time (i.e., experimental duration) strongly impact net effects and influence the difference in cumulative consumption between infected and uninfected snails. We stress that our study illustrates a powerful conceptual and mathematical approach. Despite low sample sizes for survivorship analyses, we were still able to detect parasite effects due to their strong effect sizes. Other systems that utilize this approach may need larger samples to estimate effects, if they are more subtle than the ones found here.

### 4.1 Estimating feeding rates and survivorship

In our experiment, infected snails ate twice as much as uninfected snails and had significantly lower survivorship over time. Both of these effects were influenced by temperature. Parasites have been shown to both increase (e.g., Bernot et al., 2008) or decrease consumption (e.g., Wood et al., 2007), depending on the system, and the magnitude and direction of our results align with other Planorbid snails (Muñoz et al., 2018). The increased consumption by snails (both infected and uninfected) at higher temperatures concurs with the increasing metabolic requirements of ectotherms (Schmidt‐Nielson, 1997; Willmer et al., 2000). Additionally, we found that parasite‐induced mortality increased at higher temperature treatments, aligning with previous research on marine snails (Fredensborg et al., 2005), and suggesting that high temperature functions as an additional stressor on snail hosts along with infection. Future work should include additional temperature treatments to construct temperature performance curves of feeding and mortality rates for infected and uninfected hosts (Byers, 2020, 2021; Gehman et al., 2018). This work would allow us to better predict how parasites may impact their hosts (and indirectly the larger community) due to climate change.

Digenetic trematode parasites are parasitic castrators of their first intermediate hosts that generally limit their site of infection in the host to within the gonadal region, and thus are generally considered to be nonlethal and have low parasite‐induced mortality on their hosts (Lafferty & Kuris, 2002). Our results, combined with previous studies, suggest that this may not be the case for trematodes infecting short‐lived first‐intermediate hosts, such as *Helisoma trivolvis*. Previous laboratory studies show elevated mortality rates of infected planorbid snails (Sorensen & Minchella, 2001), and field studies of *H. trivolvis* suggest midsummer parasite‐induced mortality (Peterson, 2007). For snails that live 1–2 years, such as *H. trivolvis* (Norton & Newman, 2016), given that the scope for host longevity is already so short, high lethality of infection could result because there may be less selection for trematodes to be nonlethal, or more selection for trematodes to maximize reproductive output before the host dies (Lafferty & Kuris, 2009).

### 4.2 Calculating and comparing lethal and non‐lethal effects

Using these experimental data, we found that lethal and nonlethal effects of parasites on host consumption acted simultaneously in opposing directions in our system. However, the magnitude and direction of these effects was temperature dependent. Nonlethal effects of the parasite (e.g., host feeding rate) increased resource consumption by the host and this effect increased at higher temperatures, while lethal effects decreased resource consumption and were strongest at 24°C. It is unclear why mortality peaked at our intermediate temperature, but may be an artifact of small sample sizes for estimating mortality across temperatures. Combining lethal and nonlethal effects, we found that parasites had positive net effects on resource consumption at 18°C and 30°C, but not at 24°C. Findings such as these suggest that the net impacts of parasites on resources may vary seasonally and could be impacted by climate change. Although increased host resource consumption due to parasitism is relatively rare in the literature (see Buck & Ripple, 2017), previous work has shown that parasitic castrators can increase (Bernot, 2013) and decrease host consumption of resources (Morton & Silliman, 2020; Wood et al., 2007), resulting in negative and positive indirect effects on resource populations through their snail first intermediate hosts.

### 4.3 Investigating importance of experimental duration

Our analysis of experimental duration demonstrated that the impact of parasites on total consumption changed both in magnitude and direction over time. On short time scales, most infected snails were alive and consuming twice as much as uninfected snails (nonlethal effects > lethal effects). However, as the experimental duration extended, infected snail mortality increased and the time since death (i.e., the duration of time when infected snails were not contributing towards total consumption) also increased, allowing uninfected snails to slowly overcome infected snails in total resource consumption (Figure 2d).

### 4.4 Adapting predator–prey equations

The quantitative method we have used here was adapted from those used to study TMIE and DMIE in predator–prey systems (Okuyama & Bolker, 2007). In predator–prey systems, nonlethal effects (and associated trait‐mediated indirect effects on a resource) occur primarily through prey avoidance behaviors or fear responses. Experiments generally measure the change in a basal resource (such as algae) when groups of prey are exposed to different predator treatments, including true predator (fn), threat‐only (nonlethal) predator (fN), culled prey (simulated predation) (Fn), and no predator (FN) (Okuyama & Bolker, 2007; Peacor & Werner, 2001, Preisser et al., 2005), where f and F are the per‐forager consumption in the presence and absence of predators, and *n* and *N* are the average numbers of foragers alive in the presence and absence of predation (Okuyama & Bolker, 2007). For example, in one set of studies (Beckerman et al., 1997; Schmitz, 1998; Schmitz et al., 1997), Schmitz and colleagues measured the impacts of spiders on grass through trait and density‐mediated effects on grasshoppers. To do this, they glued spiders' mouthparts shut for the threat‐only predator treatment. These treatment effects are then compared to estimate trait‐mediated (threat‐only treatment – no predator treatment, fN – FN = N(f–F)) and density‐mediated indirect effects (true predator treatment – threat‐only treatment, fn – fN = f(n–N)). Schmitz and colleagues found that trait‐mediated indirect effects were large in this system, as grasshoppers switched from eating grass to eating forbs (a safer but less caloric food source) when spiders were present.

This theory provided the solid basis for our modification to explore the holistic impacts of parasites. We have modified these equations and the methodology used in predator systems in two ways. First, because parasites (as defined by Lafferty & Kuris, 2002) attack only one host during a given life stage, whereas predators generally attack multiple prey, we estimated effects of parasites at the individual host level rather than group or population level, which is the level typically used for estimating indirect effects of predators. This means that instead of estimating the average number of prey alive at the end of an experiment of 65 days in length (*n* and *N* in Okuyama & Bolker, 2007), we estimated the number of days a host survives, and we use the variables $\bar{S\_U_{t}}$ and $\bar{S\_I_{t}}$ for survival duration of uninfected and infected hosts. Second, relatedly, predator studies measure fn, FN, etc. as inseparable units that have to be subtracted and combined to determine the isolated DMIE and TMIE. In our study, we directly estimated individual, host‐level parameter values (in our case $\bar{F\_I_{t}}$, $\bar{F\_U_{t}},\bar{S\_I_{t}},\bar{S\_U_{t}})$ to calculate average lethal ($\bar{F\_I_{t}}\left(\bar{S\_I_{t}}-\bar{S\_U_{t}}\right)$) and nonlethal effects ($\bar{S\_U_{t}}\left(\bar{F\_I_{t}}-\bar{F\_U_{t}}\right)$) rather than by comparing treatments (e.g., threat‐only treatment – no predator treatment).

### 4.5 Caveats and future directions

Here, we measured lethal (i.e., mortality) and nonlethal (i.e., feeding rate) effects in a controlled lab experiment. However, in nature there are other factors that can contribute and even interact with LE and NLE. For example, parasite avoidance behaviors may alter host foraging behavior and thus consumption in the presence of parasites (Daversa et al., 2021; Koprivnikar et al., 2021; Weinstein et al., 2018), as has been shown for prey in the presence of their predators (e.g., Peckarsky et al., 2008; Werner & Peacor, 2003). Despite limited studies on parasite avoidance behaviors in snails, evidence suggests that uninfected snails do perceive parasite cues in the environment and respond by avoiding them (Davies & Knowles, 2001). This could cause snails to decrease foraging, which is a common route of infection, and would decrease the relative importance of nonlethal versus lethal effects in this system and should be considered in future work. Infection may also cause additional lethal effects, such as increased vulnerability to predation (e.g., healthy herd hypothesis; Gehman & Byers, 2017). This would further strengthen the relative role of LE compared with NLE. Factors such as these were disallowed in our controlled lab experiment, but may be influential in natural settings.

One caveat to investigating lethal and nonlethal effects at a per‐capita basis is that we cannot investigate how changes in snail density influence per‐capita feeding rate or mortality rates when resources are limiting. In our experiment with individually housed snails, and thus no intraspecific resource limitation, snails may have had higher per‐capita feeding rates and lower mortality rates than they would have had under higher density conditions, due to lack of competition. However, a review by Sorenson and Minchella (Sorensen & Minchella, 2001) of 113 studies found that infected snails actually had higher mortality than uninfected snails more often when isolated than when grouped (93% vs 50% of the time), suggesting that lethal effects of parasites could be lower when in a group setting (Sorensen & Minchella, 2001). However, while the mechanism of this effect is unknown, it may be an artifact of experimental infection: individually housed snails may end up with higher infection doses (e.g., more miracidiae) than snails housed in groups.

In this study, we use a nonliving food item (blended food agar) as a proxy for a diverse assemblage of basal resources (periphyton, detritus, etc). Thus, the resource has no productivity and cannot increase, for example, if grazing diminishes. However, our quantitative framework for comparing lethal and nonlethal effects could be adapted to a dynamic, i.e., living, basal resource. In fact, if the resource is living, and thus capable of dynamic feedback and growth, it presents the opportunity to investigate the relative roles of density‐mediated indirect effects and consumptive trait‐mediated indirect effects on basal resource dynamics (sensu Buck, 2019). In these instances, one would be measuring the standing stock and productivity of the resource, as opposed to the feeding rate of the host on that resource as we did here. Additionally, since we would be looking at the impact on the resource, rather than amount consumed, increased feeding rates by infected individuals should cause negative trait‐mediated indirect effects and increased mortality rates by infected individuals should cause positive density‐mediated indirect effects. Thus, the same equations could be used with only slight sign modifications: trait‐mediated indirect effects = −$\bar{S\_U_{t}}\left(\bar{F\_I_{t}}-\bar{F\_U_{t}}\right)$ and density‐mediated indirect effects = −$\bar{F\_I_{t}}\left(\bar{S\_I_{t}}-\bar{S\_U_{t}}\right)$, with $\bar{F\_I_{t}}$ and $\bar{F\_U_{t}}$ being the change in resource per unit time due to an infected or uninfected snail, respectively.

## 5 CONCLUSIONS

Lethal and nonlethal effects of parasites can contribute significantly to ecosystems (Buck, 2019), trophic cascades (Buck & Ripple, 2017), and invasions (Dunn et al., 2012). Altogether, this work provides a novel framework for calculating lethal and nonlethal effects of parasites and demonstrates their relative importance for host resource consumption. Importantly, it illustrates that lethal and nonlethal effects of parasites can oppose each other. This is unusual in the predator–prey literature, where nonlethal effects on prey (e.g., avoidance behaviors) typically reduce resource consumption, and thus act in concert with lethal effects on prey, which also reduce resource consumption. The opposing direction of the parasite effects on hosts suggests that parasites can potentially increase net consumption of basal resources by their hosts. In such instances, we may expect that host populations with higher infection prevalence (given the same host density) should exhibit stronger top‐down control over basal resources than uninfected host populations. Conversely, other snail species (namely longer lived marine taxa) show decreased feeding rates when infected with a trematode, resulting in snail host populations exhibiting less top‐down control. In both cases, where snail hosts are dominant or keystone grazers, the effects of parasites could affect food web structure, by changing resource availability and interspecific competition. However, the direction in which trematodes influence food web structure crucially depends on the joint impacts of lethal and nonlethal effects. Furthermore, the sensitivity of parasite‐mediated effects to temperature and experimental duration that we demonstrated, emphasize careful, holistic consideration of the lethal and nonlethal effects of parasites.

## AUTHOR CONTRIBUTIONS

**Emlyn J. Resetarits:** Conceptualization (equal); data curation (lead); formal analysis (lead); investigation (lead); methodology (equal); supervision (lead); visualization (lead); writing – original draft (lead); writing – review and editing (equal). **William T. Ellis:** Formal analysis (supporting); investigation (equal); writing – original draft (supporting). **James E. Byers:** Conceptualization (equal); formal analysis (supporting); investigation (equal); methodology (equal); writing – original draft (supporting); writing – review and editing (equal).

## CONFLICT OF INTEREST STATEMENT

The authors declare that they have no conflicts of interest.

## Supporting information


**Appendix S1:** Supporting Information

## Data Availability

The datasets used and/or analyzed during the current study are available from the corresponding author on reasonable request. The data that support the findings of this study will be available on github (URL TBD) upon publication.
