# Supplementary material for: The opposing roles of lethal and nonlethal effects of parasites on host resource consumption
Source: Ecol Evol. 2023 Apr 13;13(4):e9973. doi: 10.1002/ece3.9973 (PMC10099202; doi:10.1002/ece3.9973)
Supplement: Supplementary file 1 — Appendix S1: Supporting Information [file ECE3-13-e9973-s001.pdf]

Supplementary Materials

Title: The opposing roles of lethal and non-lethal effects of parasites on host resource consumption

Below are supplementary materials regarding equations 1-3.

We can derive lethal effects (LE) from equations 1 and 2:

$$\overline{S\_I_t} \cdot \overline{F\_I_t} = \overline{S\_U_t} \cdot \overline{F\_U_t} + (\text{LE} + \text{NLE})$$

$$\overline{S\_I_t} \cdot \overline{F\_I_t} = \overline{S\_U_t} \cdot \overline{F\_U_t} + (\text{LE} + \overline{S\_U_t}(\overline{F\_I_t} - \overline{F\_U_t}))$$

$$\overline{S\_I_t} \cdot \overline{F\_I_t} = \overline{S\_U_t} \cdot \overline{F\_U_t} + (\text{LE} + \overline{S\_U_t} \cdot \overline{F\_I_t} - \overline{S\_U_t} \cdot \overline{F\_U_t})$$

$$\overline{S\_I_t} \cdot \overline{F\_I_t} = \overline{S\_U_t} \cdot \overline{F\_U_t} + \text{LE} - \overline{S\_U_t} \cdot \overline{F\_U_t} + \overline{S\_U_t} \cdot \overline{F\_I_t}$$

$$\overline{S\_I_t} \cdot \overline{F\_I_t} = \text{LE} + \overline{S\_U_t} \cdot \overline{F\_I_t}$$

$$\text{LE} = \overline{S\_I_t} \cdot \overline{F\_I_t} - \overline{S\_U_t} \cdot \overline{F\_I_t}$$

$$\text{LE} = \overline{F\_I_t}(\overline{S\_I_t} - \overline{S\_U_t})$$

These equations could also be written with NLE = $\overline{S\_I_t}(\overline{F\_I_t} - \overline{F\_U_t})$ and LE = $\overline{F\_U_t}(\overline{S\_I_t} - \overline{S\_U_t})$. Because there are two ways to write these equations, this makes it difficult to directly comparing absolute LE and NLE, the net consumptive effects (Net Consumptive Effects = LE + NLE) and thus the difference between LE and NLE are robust and unaffected by these differing equations.

Values used for calculating total resource consumption, net consumptive effects, lethal effects, and non-lethal effects are shown in Table S1.

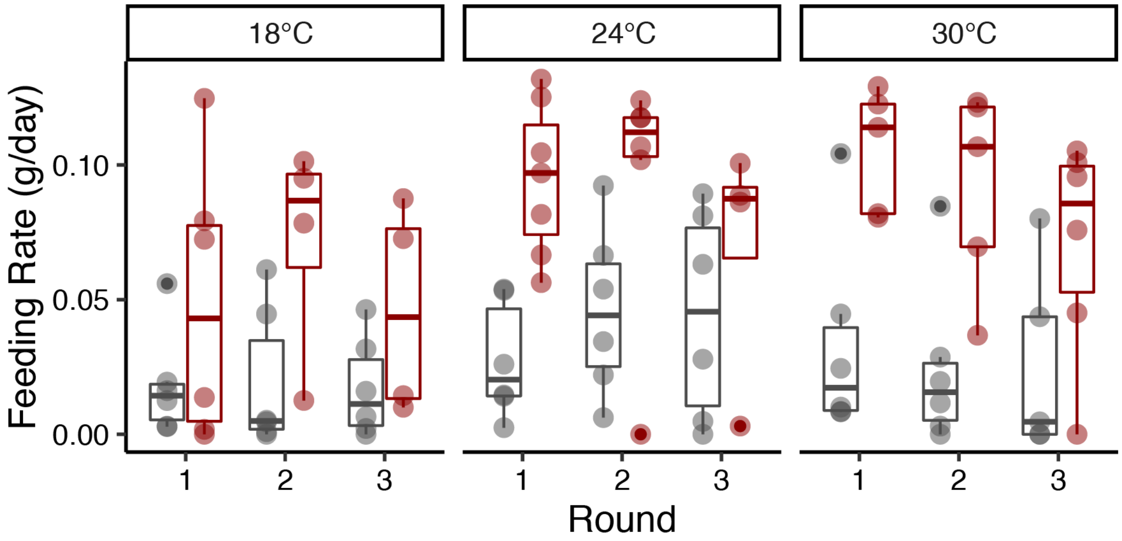

Figure S1 – Individual feeding rates (g/day) and boxplots of individual snails for three experimental trials as a function of temperature and infection status. Red points represent infected snails and black represent uninfected snails.

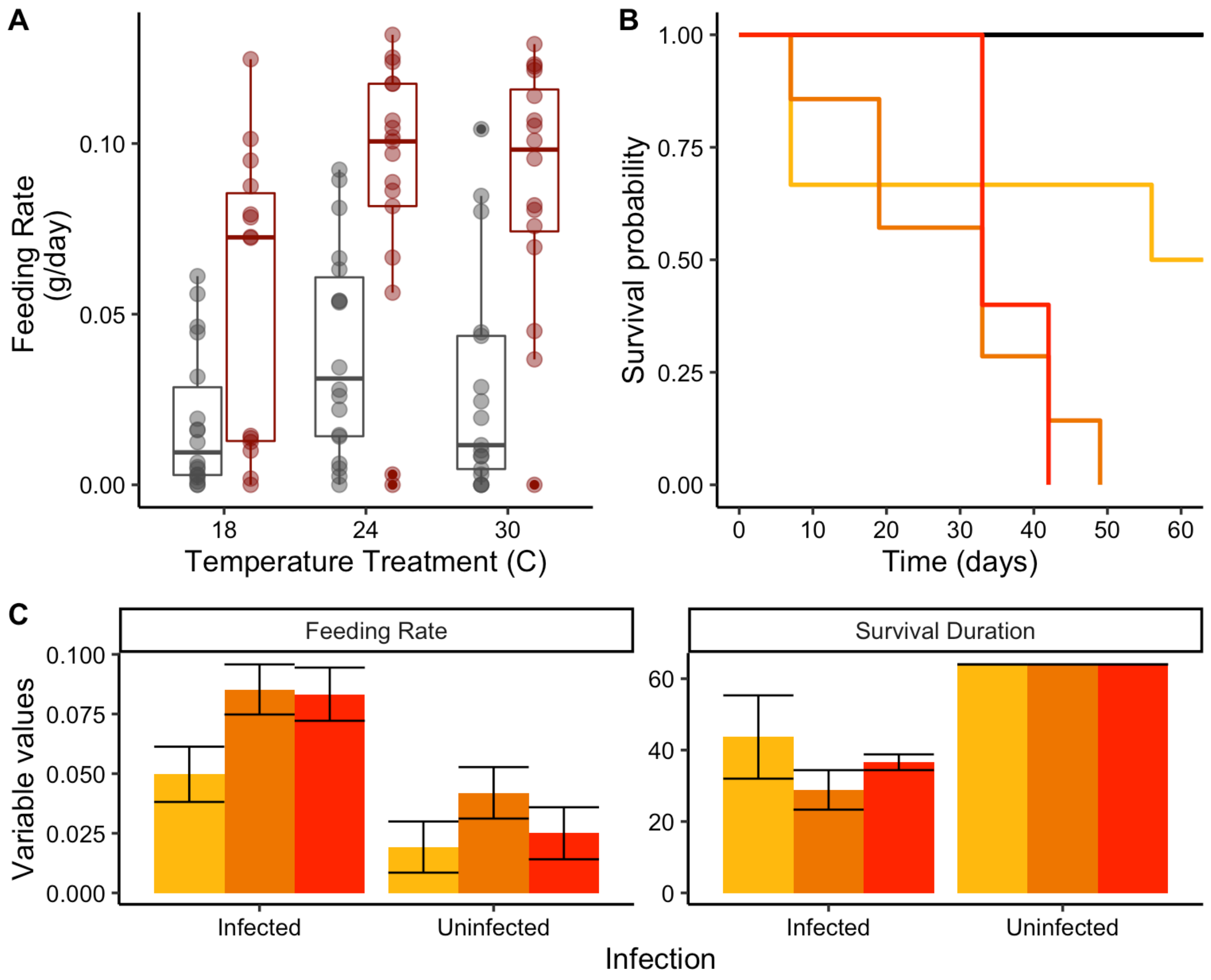

Figure S2 - A) Infection status and temperature significantly impact feeding rates. Infected snails (dark red) eat significantly more than uninfected snails (grey). B) Infection status and temperature also significantly impact survivorship. Survivorship is significantly lower for infected snails than uninfected snails. There was no mortality for uninfected snails at all temperatures (black line). Yellow = infected, 18°C; orange = infected, 24°C; red = infected, 30°C. C) Left: Estimated marginal means and standard error were used to calculate feeding rates for uninfected and infected hosts at each temperature (F_U_t and F_I_t) for calculating lethal and non-lethal effects. Right: Mean and standard error for survivorship duration at each temperature

for infected and uninfected snails (S_U_t and S_I_t) were used for calculating lethal and non-lethal effects. Yellow = infected, 18°C; orange = infected, 24°C; red = infected, 30°C.

Table S1 – Values extracted from experimental data for calculating lethal and non-lethal effects. $\overline{S\_U_t}$ and $\overline{S\_I_t}$ are the mean and standard error values of survival duration for uninfected and infected snails, respectively. $\overline{F\_U_t}$ and $\overline{F\_I_t}$ are the estimated marginal mean and standard error values for feeding rates (g/day) for uninfected and infected snails, respectively.

**Temperature Treatment (°C)**	$\overline{S\_U_t}$	$\overline{S\_I_t}$	$\overline{F\_U_t}$	$\overline{F\_I_t}$
**18**	64 ± 0.000	43.667 ± 11.664	0.019 ± 0.011	0.051 ± 0.011
**24**	64 ± 0.000	28.857 ± 5.531	0.042 ± 0.011	0.086 ± 0.010
**30**	64 ± 0.000	36.600 ± 2.205	0.025 ± 0.011	0.083 ± 0.011

Table S2 – Infected snails used in the experiment were infected with different trematode morphotypes.

Temperature Treatment (°C)	Magnacaudal morphotype	Strigea morphotype	Unidentified morphotype
**18**	1	4	1
**24**	0	7	0
**30**	1	3	2

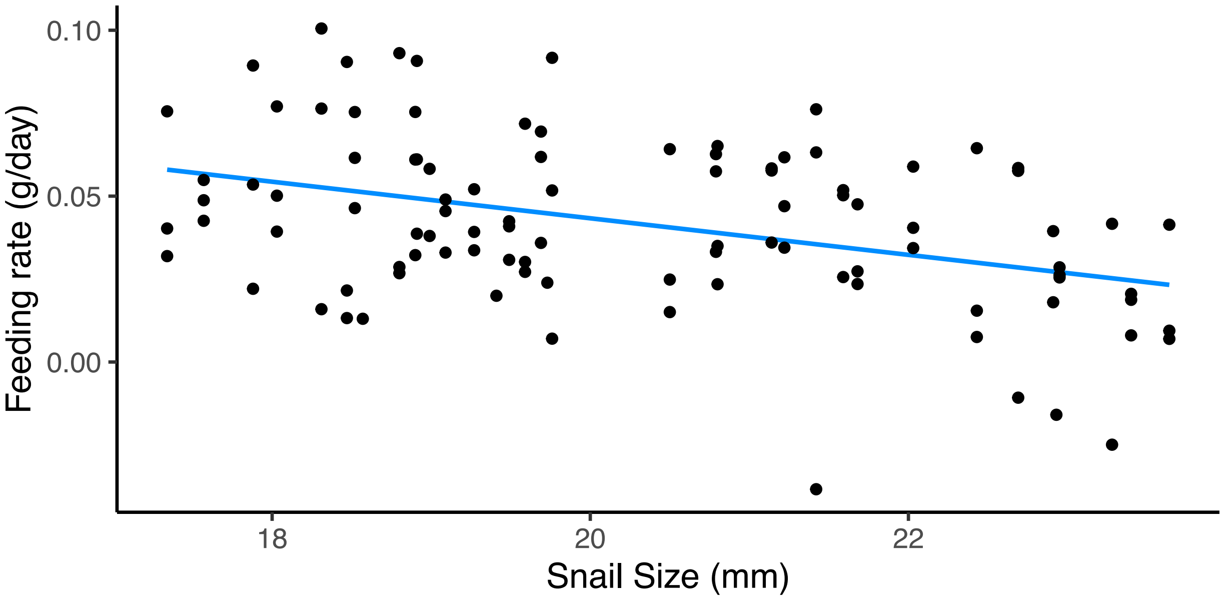

Figure S2 - Consumption decreased with snail size (χ^2 = 5.209, df = 1, p = 0.022). Effect of snail size on consumption, controlling for infection status and temperature.
